# Genetic Diversity and Metabolic Profile of Tibetan Medicinal Plant *Saussurea obvallata*

**DOI:** 10.3390/genes16050593

**Published:** 2025-05-17

**Authors:** Shengnan Zhang, Sujuan Wang, Shiyan Wang, Hao Su, Ji De

**Affiliations:** 1 Key Laboratory of Biodiversity and Environment on the Qinghai-Tibetan Plateau, Ministry of Education, Lhasa 850000, China; xunzhaodingxiang@163.com (S.Z.); wangsujuan71@stu.utibet.edu.cn (S.W.); wsy13312551226@163.com (S.W.); su1220834362@163.com (H.S.); 2 School of Ecology and Environment, Xizang University, Lhasa 850000, China

**Keywords:** *Saussurea obvallata*, genetic diversity, metabonomics

## Abstract

Background/Objectives: *Saussurea obvallata* (DC.) Edgew., Asteraceae, is a traditional medicinal herbnative to the Qinghai–Tibet Plateau (QTP). Pharmacological investigationshave validated its pharmacological effects in anti-tumor, anti-inflammatory, heat-clearing, detoxifying, and analgesia. *S. obv* is presently facing habitat fragmentation and population decline. Therefore, we analyzed its genetic and chemical diversity to provide a scientific basis for the conservation and sustainable use of *S. obv*. Methods: Seven populations of *S. obv* were sampled from Xizang, China. The genetic diversity was analyzed using inter-simple sequence repeat (ISSR) markers, and metabolites were identified by ultra-high-performance liquid chromatography-tandem-quadrupole-time-of-flight mass spectrometry (UPLC-Q-TOF-MS/MS). Correlation analysis among genetic diversity, differential metabolites, and climatic factors were performed by R. Results: The genetic diversity among and within populations were both lowly and significantly correlated with geographical distance, showing a decreasing trend from east to west of the QTP. A total of 110 compounds were identified, including flavonoids, phenylpropanoids, lipids, fatty acids, terpenoids, alkaloids, etc. The metabolite contents among populations varied greatly and were related to environmental factors, mainly annual mean temperature and temperature fluctuation. The genetic diversity had little effect on the metabolic differences. Conclusions: These findings provided valuable baseline information for the conservation and pharmacological utilization of *S. obv*. Meanwhile, further research is necessary for the efficacy evaluation of anti-inflammatory, anti-tumor, radiation protection, and scar removal both in vitro and in vivo.

## 1. Introduction

**Figure 1:**
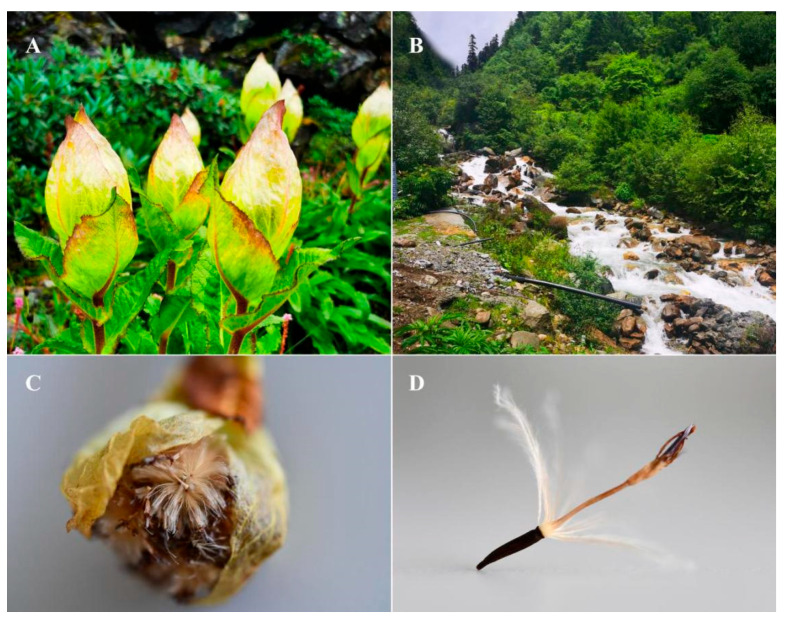
Morphology and habitat of *S. obvallata*. (**A**) The bracts forming a “greenhouse”. (**B**) Typical habitat of *S. obv*. (**C**) Bracts opening as fruits ripen. (**D**) Tubular floret, pappi, and fruit.

*Saussurea* DC. is one of the largest genera in the Asteraceae family, with more than 500 species found in the northern hemisphere [1]. *S. obvallata* (DC.) Edgew., a perennial hermaphrodite herb of the Asteraceae family, is endemic to the Qinghai–Tibet Plateau (QTP), thriving at altitudes between 3200 and 4700 masl. *S. obv* is commonly found in alpine screes, alpine meadows, rocky slopes, shrubs, and near streams [2,3]. The stones and shrubs can shield *S. obv* from strong wind, allowing it to achieve an average height of 50–80 cm. *S. obv* blooms from July to September. It is a typical “greenhouse plant”. The light-yellow bracts envelop flowers, thus protecting them from UV light and retaining heat at night (Figure 1A), attracting insects for cross-pollination. By September and October, the fruits will gradually mature, and the bracts will open (Figure 1C). The fruits with pappi (Figure 1D) are dispersed by wind and grow in suitable habitats [4]. During winter, the above-ground parts wither, and the roots will regenerate in the following spring [5].

Over the past years, global climate change and human alterations have causedhabitat fragmentation and a dramatic decline in distribution. In 2009, the Science and Technology Department of Xizang Autonomous Region classified it as a Class III endangered medicinal plant [6]. In 2017, Fei et al. investigated the medicinal plant resources of Sejira Mountain, and *S. obv* was suggested as a Class II endangered species [7]. In India, because of its wide application in medicine, decoration, and social economy [8], coupled with the weak ecological awareness of residents and tourists, *S. obv* is facing over-harvesting and, therefore, has been listed as an endangered species and one of the highest priorities for conservation programs by the Conservation Assessment Management Plan (CAMP) and Convention of International Trade in Endangered Species of Wild Fauna and Flora (CITES).

More than 40 *S.* species have been used as traditional medicinal plants in China [9,10,11], and their phytochemicals and pharmacological activities havealso been investigated for many years [12,13]. The main compounds identified from *S.* species are terpenoids, flavonoids, lignans phenylpropanoids, and polysaccharides, and they were mainly used for anti-tumor, antibacterial, anti-inflammatory, and anti-ulcer properties. *S. medusa*, containing neoechinulin A, arctigenin, epigenin, and luteolin, has been used to protect patients from inflammation, ultraviolet radiation, cold, hypoxia, and skin photo-aging [14,15,16]. *S. costus* is an endangered species, traditionally used for a variety of diseases, showing potent effects on carminative, expectorant, anti-arthritic, and antiseptic [17]. *S. involucrata*, mainly distributed in Xinjiang, China, was found to have neuroprotection effect, ischemia/reperfusion injury protection, adipogenesis suppression, anti-tumor effect, anti-inflammation, cardio-protective effect, and other pharmaceutical values [18,19].

Due to its antipyretic, antidotal, anti-inflammatory, and analgesic effects, *S. obv* has been used as a traditional medical plant in China, India, and Pakistan [20,21,22,23,24]. It is recorded in ancient Tibetan medicine books “The Four Medical Tantras”, “Crystal Materia Medica”, “The Stainless Crystal Mirror: A Tibetan Materia Medica”, and so on. Over 130 phytochemicals have been reported in *S. obv*, including flavonoids, terpenoids, fatty acids, and alkaloids [25,26,27,28,29,30,31,32,33]. These metabolites not only play an important role in plant response to environmental stressbut also are the pharmacological basis of medicinal plants.

Metabolomics studies all metabolites in the biological body. Compared with genomics and proteomics, it reflects the immediate biochemical state in the biological body. In addition, metabolomics can provide comprehensive information about the types and contents of various metabolites and quickly obtain the changes of metabolites and their change rules in different environments, different growth periods, different parts, or under certain specific stimuli [34,35]. Metabolomics technology has been widely used because of its relatively low price and labor-saving [36].

The primary metabolites of plants, such as proteins, lipids, and polysaccharides, are essential substances to maintain basic life activities. Secondary metabolites, including flavonoids, terpenes, alkaloids, coumarins, and other small-molecule compounds, not only play a key role in plant stress responses, such as UV protection, antibacterial, and resistance to pests and diseases, but also have important value for human health [37].

UPLC-Q-TOF-MS/MS is a liquid chromatography-mass spectrometry combined technology, which has high sensitivity, good separation effect, fast detection speed, and relatively accurate mass charge ratio for detected ions.Therefore, it is widely used in natural product analysis and metabolomics research [38,39].

In this study, the ISSR marker was used to assesspopulation genetic variation, and UPLC-Q-TOF-MS/MS was used to determinethe metabolism. Analyzing the genetic diversity and differential metabolites of different populationsprovidesbaseline data for the conservation, development, and utilization of *S. obv*.

## 2. Materials and Methods

### 2.1. Population Sampling and Specimen

**Figure 2:**
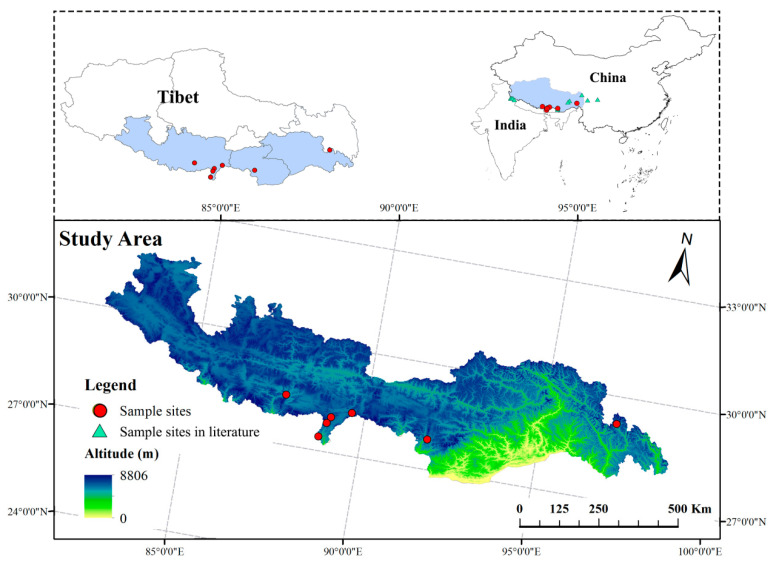
Sampling sites map of seven populations of *S. obv*.

Seven genetically distinctpopulations of *S. obv* were sampled across the Xizang Autonomous Region from July–August 2022, maintaining minimum interpopulation distances >50 km andintrapopulation sampling intervals > 20 m to minimize kinship bias, as illustrated in Figure 2. Figure 2 also indicatesthe locations of *S. obv* sampling sites reported in the previous literature [40,41,42,43]. Detailed geographical characteristics were described in Table 1. Compared with Lhasa, the habitats of *S. obv* are much more humid. There are streams flowing near the habitats. The communities of all seven sapling sites are similar, mainly composed of *Rhododendron* spp., *Rhodiola rosea*, *Dasiphorafruticosa*, *MeconopsisVig.*, *Bistorta macrophylla*, and other plants.

For genetic diversity analyses, 20 tender leaf samplesper population underwentsilica gel-mediated rapid desiccation. Concurrently, six phenotypically representative aerial modules (stems, leaves, inflorescence) per site were collected for metabolomic studies andshade-dried. At each site, a complete individual plant was collected and preserved as a voucher specimen. The corresponding specimen numbers are listed in Table 1. All specimens are deposited in the School of Ecology and Environment, Xizang University, Lasa, Xizang Autonomous Region, China.

### 2.2. Climatic Data

Climatic data were obtained from National Tibetan Plateau Data Center/Third Pole Environment Data Center [44] and National Earth System Science Data Center [45].

### 2.3. DNA Extraction and PCR Amplification

Cryogrinding of dried leaf tissues was performed using Sample Preparation System (FastPrep-24^TM^, MP Biomedicals, Santa Ana, CA, USA), and total genomic DNA was extracted using Plant Genomic DNA Extraction Kit (D1500, Solarbio, Beijing, China). A hundred universal ISSR primers published by the University of British Columbia (Table S1) were synthesized by Sangon Biotech Co., Ltd. (Shanghai, China). All primers were screened, and those with high polymorphism were selected for genetic diversity analysis.

The polymerase chain reaction (PCR) was conducted on all 140 samples in thermal cyclers (T100^TM^, BIO-RAD, Hercules, CA, USA), in a total volume of 25 μL containing 1 μL of DNA template, 2 μL of primer, 9.5 μL of ddH_2_O, and 12.5 μL of Taq PCR Mastermix (PC1150, Solarbio, Beijing, China). The PCR amplification procedures were set as Table S2.

All PCR products were checked using 2% agarose gel containing 0.1% nucleic acid dye (G5560, Solarbio, Beijing, China), and photos were taken under UV light (ChemiDoc XRS System, BIO-RAD, Hercules, CA, USA).

### 2.4. Data Analysis

Boolean matrix was used to analyze genetic diversity. According to the migration of PCR products, each band was considered as a DNA marker and scored across all samples. The present and clear bands were recorded as 1, and the absent or faint bands were recorded as 0. The percentage of polymorphic bands (PPL), number of observed alleles (Na), number of effective alleles (Ne), Nei’s gene diversity (H), and Shannon’s information index (I) were calculated using POPGENE32 v1.32 programme. Analysis of molecular variance (AMOVA) and Mantel test were calculated using GenAlEx 6.503 programme. The correlation analysis between genetic diversity and climatic factors was performed with R4.3.3programme [46].

### 2.5. Preparation of Extract

The dried aerial parts of *S. obv* were powdered using a grinder (FSJ-A03D1, Bear, Foshan, China) and sieved. A half a gram of powder was weighed (ME204E, METTLER TOLEDO, Greifensee, Switzerland) and ultrasound-treated separately with 10 mL of 80% ethanol for 30 min (KQ3200E, Supmile, Shanghai, China). The supernatant was filtered with a 0.22 μm filter membrane (JTSF Nylon66, JINTENG, Zhangjiagang, China) and stored at 4 °C. Twenty-five microliters of each sample were mixed as Quality Control (QC) solution.

### 2.6. Identification of Metabolites

The metabolites of ethanol extracts were identified and analyzed using ultra-high-performance liquid chromatography-tandem quadrupole-time-of-flight mass spectrometry system (UPLC-Q-TOF-MS/MS) (Exion LC, Shimadzu, Kyoto, Japan; X500R, SCIEX, Framingham, MA, USA) and a C18 column (2.1 × 100 mm, 1.7 µm, ACQUITY UPLC^®^ BEH, Waters, Milford, MA, USA). The oven temperature was set at 30 °C; the mobile phase A was acetonitrile, and the mobile phase B was 0.1% formic acid aqueous solution; the flow rate was 0.3 mL/min, and the injection volume was 5 µL. The eluent gradient was set at Table S3. The mass parameters were set as Table S4.

### 2.7. Analysis of Metabolites

The extraction samples and QC solution were analyzed in accordance with the conditions of 2.6. The data-acquisition and processing software was SCIEX OS v3.3.1.43 software and was delivered with the instrument. According to primary and secondary mass spectrometry information, metabolites were identified using the natural product database of SCIEX OS software and the GNPS natural product database of MS-DIAL v2.64 software. Differential metabolite analysis was performed using the MetaboAnalyst platform (https://www.metaboanalyst.ca, accessed on 18 March 2025), and correlation analyses were conducted using R4.3.3.

Principal Component Analysis (PCA) is an unsupervised analysis method that visualizes complex data through dimensional reduction. Therefore, PCA was used to show the metabolic differences amongthe seven populations of *S. obv*.

*p* values of *t*-test < 0.05, fold change > 2 or <0.5, and PLS-DA VIP > 1 were used as criteria for differential metabolites screening. Foldchange (FC) analysis was used to compare the absolute value of metabolic change between two group means. Partial Least-Squares Discriminant Analysis (PLS-DA) is a supervised dimensional reduction method that incorporates class labels in the analysis. It captures the variance and correlation structure in the data most relevant to group differentiation. Variable Importance in Projection (VIP) is a feature that ranks the metabolites based on their significance in the PLS-DA model’s ability to discriminate between different groups.

Differential metabolites were annotated using the HMDB database (https://hmdb.ca/, accessed on 18 March 2025), KEGG database (https://www.genome.jp/kegg/, accessed on 18 March 2025), and LIPID MAPS database (https://lipidmaps.org/, accessed on 18 March 2025).

KEGG pathway enrichment and topology analysis were conducted on the differential metabolites of all comparable groups of *S. obv*. The *p* value indicates the extentof pathway enrichment;the smaller the *p* value, the greater the difference inenrichment. Pathway Impact refers to the role of metabolites in the pathway, and the greater the pathway impact, the greater the role of metabolites in the pathway. Therefore, the pathway with the larger −log10(p) value and the larger pathway impact would be selected as the significantly enriched pathway.

## 3. Results

### 3.1. Primer Screening and PCR Products

**Figure 3:**
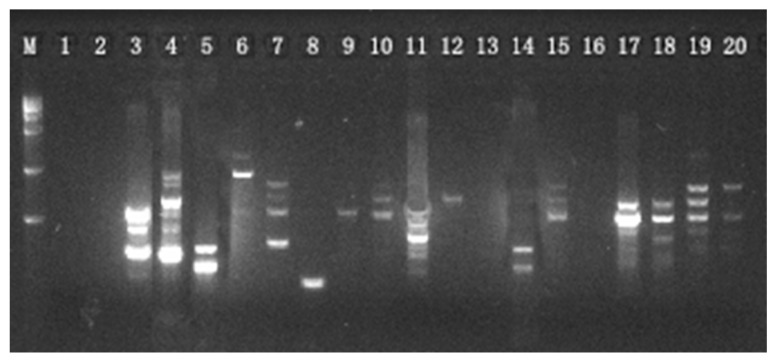
Amplification of Primer 808 from 20 samples of Population 6.

A total of 20 primers were selected and further used for the genetic analysis of *S. obv*. The characteristics of the 20 primers are listed in Table 2. Agarose electrophoresis revealed 185 ISSR loci across 140 samples exhibitingcomplete polymorphism (Rate of Polymorphic Bands = 100%).Representative electropherogram profiles of primer 808 in Population 6 (Figure 3) demonstrate banding patterns.

### 3.2. Genetic Diversity Analysis of 7 Populations of S. obv

Analyses of genetic diversity within each population of *S. obv* were reported in Table 3. The highest level of polymorphism was observed in Population 7 (PPL = 90.3%) and the lowest in Population 5 (PPL = 45.6%). The highest number of observed alleles was found in Population 7 (Na = 1.9029) and the lowest in Population 5 (Na = 1.4563). The highest number of effective alleles was found in Population 6 (Ne = 1.4439), and the lowest in Population 5 (Ne = 1.1122). Population 6 showed the maximum Nei’s gene diversity (H = 0.2698) and the maximum Shannon’s information index (I = 0.4145), while Population 5 showed the minimum Nei’s gene diversity (H = 0.0834) and the minimum Shannon’s information index (I = 0.1454). Generally, Populations 6 and 7 showed the highest genetic diversity, and Population 5 showed the lowest genetic diversity.

Among seven populations of *S. obv*, the number of observed alleles (Na) was 1.8333, and the number of effective alleles (Ne) was 1.5093. Nei’s gene diversity (H = 0.3027) and Shannon’s information index (I = 0.4535) indicated certain genetic variability among populations.

AMOVA showed that the genetic differentiation index (Fst) of seven populations was 0.114 (*p* < 0.001). The mean inter-population variation was 0.913, accounting for 11% of the total genetic variation in the sample. The mean within-populationvariation was 7.704, accounting for 89% of the total genetic variation, indicating that only 11% of all genetic variation was caused by inter-population differences, further confirming that the genetic differentiation among populations was relatively small, and the level of genetic variation within populations was much larger. The results were shown in Table 4.

**Figure 4:**
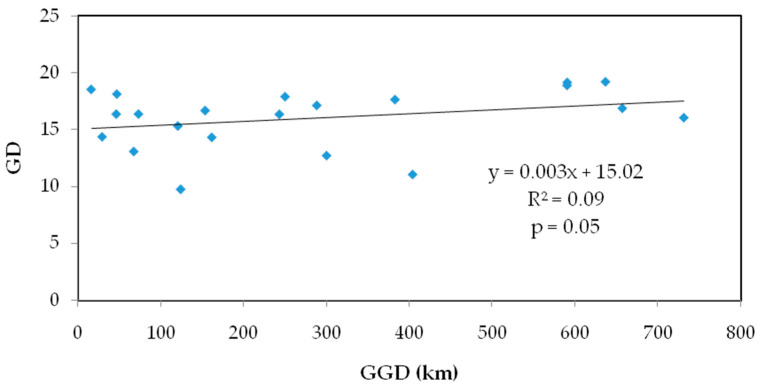
Mantel test on genetic distance and geographic distance of seven populations of *S. obv*.

The values of seven populations of *S. obv* ranged from 0.4583 to 0.8167 (Table 5), only one of which was below 0.5, indicating low genetic differentiation among *S. obv* populations. Mantel test showed that there was a significant correlation between genetic distance and geographical distance (Figure 4).

### 3.3. Correlation Analysis Between Genetic Diversity and Climatic Factors

The climatic factors of the seven sampling sites were recorded in Table 6. According to the sampling loci information (Table 1), the altitudes are between 4000–4500 m.

**Figure 5:**
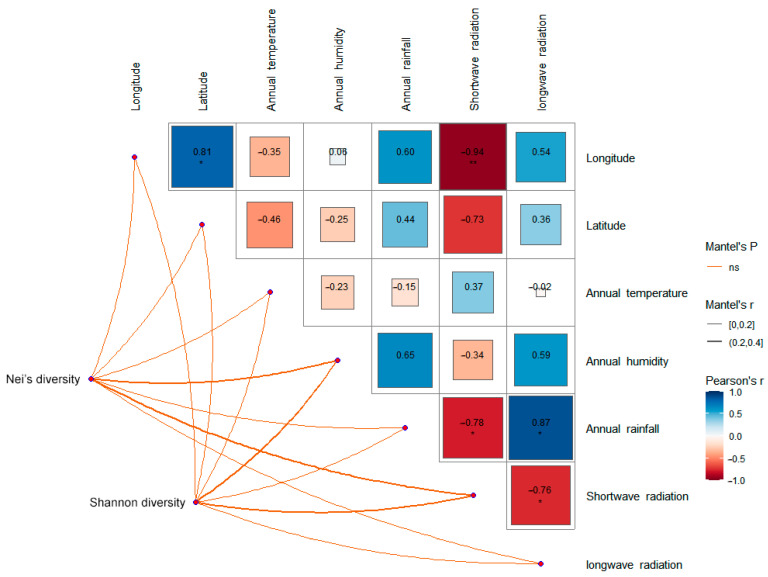
Correlation analysis between genetic diversity and climatic factors of seven populations of *S. obv*. * *p* < 0.05, ** *p* < 0.01.

The climate of the QTP is relatively stable, and the higher temperature and precipitation mostly appear from June to September, which is also an important period for the growth and propagation of *S. obvallata*. The average annual temperature of the seven sampling sites was in the range of 0 ± 2 °C. The average annual moisture content was in the range of 4–8‰. The average annual precipitation was in the range of 400–830 mm. The short-wave radiation was in the range of 174.22–236.87 W/m^2^. The long-wave radiation was in the range of 232.37–283.36 W/m^2^. Correlation analysis showed that there was no significant correlation between genetic diversity and the above climatic factors (Figure 5).

### 3.4. Identification of Ethanol Extracts of S. obv

**Figure 6:**
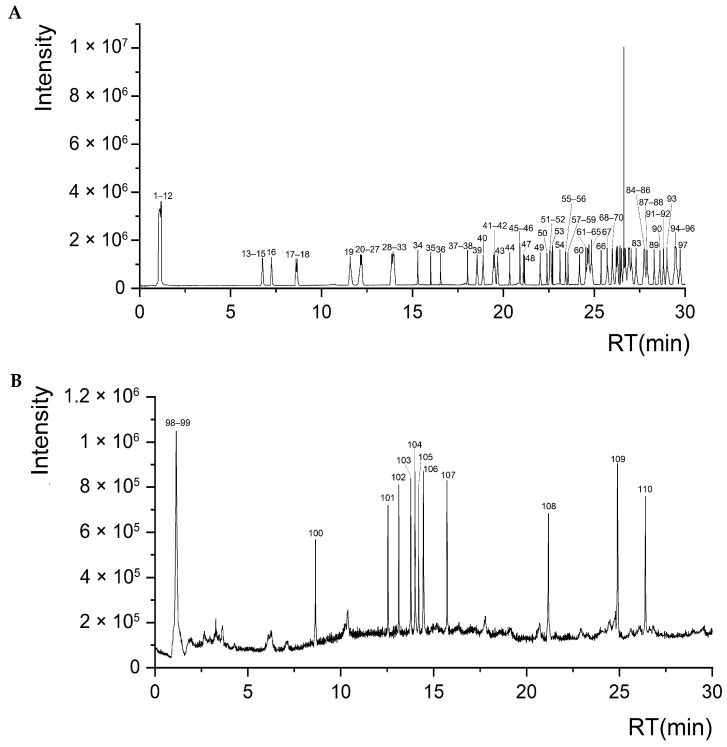
Total ion chromatograms of *S. obv* ethanol extract. (**A**) Positive ion mode. (**B**) Negative ion mode.

Mass Error < 5%, Difference Isotope Ratio < 5%, and Library Hit Score > 70 were used as criteria for screening, and 110 compounds were identified from the ethanol extracts of *S. obv* (Table S5), including 25 flavonoids, 15 phenylpropanoids, 12 lipids, 10 fatty acids, 7 terpenoids, 5 alkaloids, and other components. Figure 6 shows the total ion chromatograms in positive and negative ion modes.

### 3.5. Differential Metabolites Among 7 Populations of S. obv

**Figure 7:**
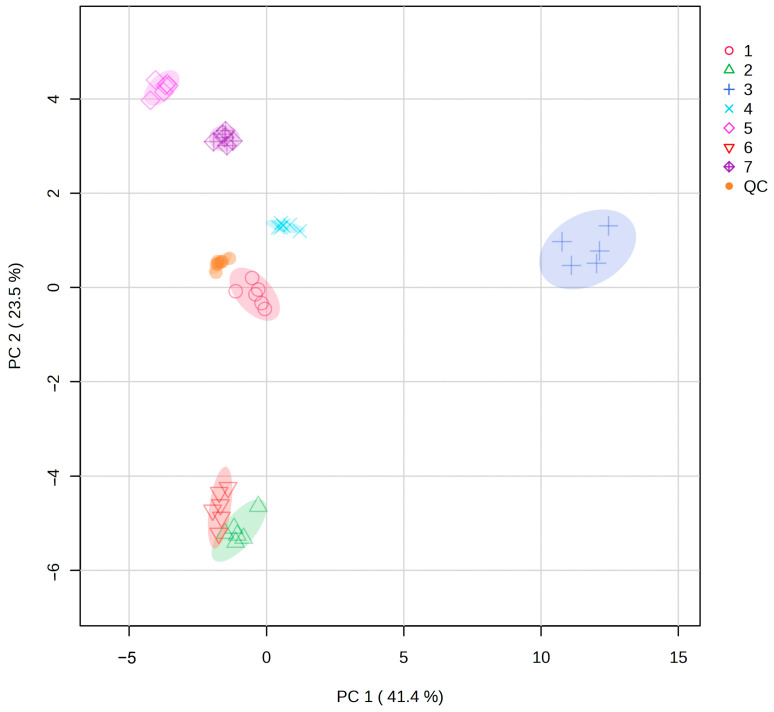
PCA of metabolites from seven populations of *S. obv*.

Metabolites of different populations of *S. obv* were analyzed by PCA, and the results areshown in Figure 7. All QC samples were gathered together, indicating the reliability of the results. The samples within the population showed good reproducibility, while most inter-population samples demonstrated clear separation in metabolite profiles, except for a minor overlap between Population 2 and Population 6.

**Figure 8:**
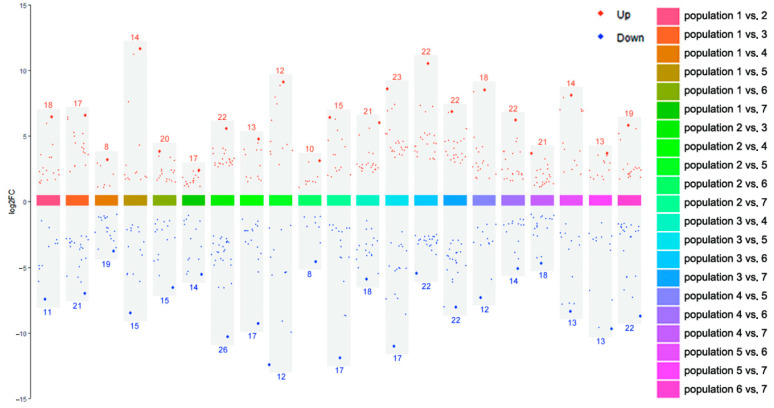
Differential metabolites between every two populations of *S. obv*. Note: The red/blue numbers are the number of upregulated/downregulated differential metabolites in each comparable group.

*p* values < 0.05, Fold change > 2 or <0.5, and PLS-DA VIP > 1 were used as criteria for differential metabolites screening. Of all 21 comparable groups, 85 metabolites were significantly different in at least one comparable group (Table S6). Arctiin, curdionolide B, resedine, and querciturone differed significantly in 17 comparable groups; arctigenin, betaine and 8-hydroxycadalene differed significantly in 16 comparable groups; isoalantolactone and dehydrocostus lactone differed significantly in 15 comparable groups. The differential metabolites between all comparable groups are shown in Figure 8. The upregulated differential metabolites ranged from 8 to 23, and the downregulated differential metabolites ranged from 8 to 26. The upregulation of arctiin was the highest (log_2_FC = 11.559, Population 1 vs. Population 5), and the downregulation of curdionolide B was the highest (log_2_FC = −12.486, Population 2 vs. Population 5).

### 3.6. Annotation of Differential Metabolites

**Figure 9:**
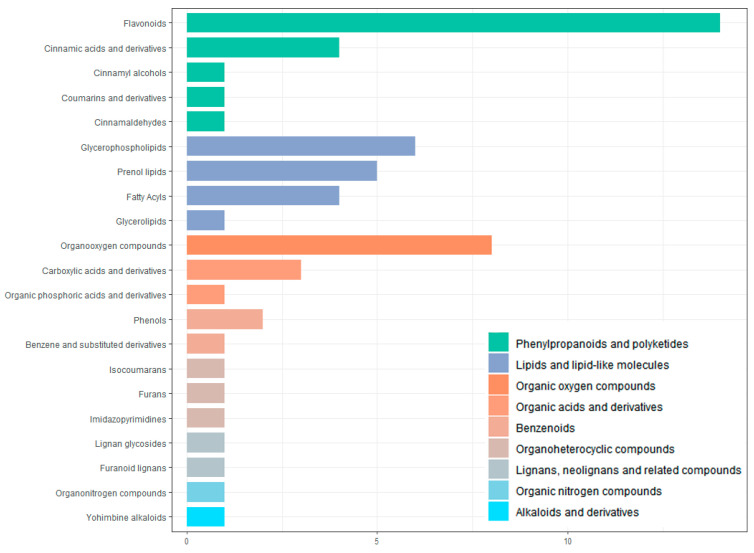
Classification of differential metabolites by HMDB database annotation.

All screened differential metabolites were annotated to the HMDB database (Figure 9). The metabolites were divided into nine categories, including phenylpropanoids and polyketides, lipids and lipid-like molecules, organic oxygen compounds, organic acids and derivative, benzenoids, organoheterocyclic compounds, lignans, neolignans and related compounds, organic nitrogen compounds, alkaloids, and derivatives. The largest number of metabolites is flavonoids (14 in total).

**Figure 10:**
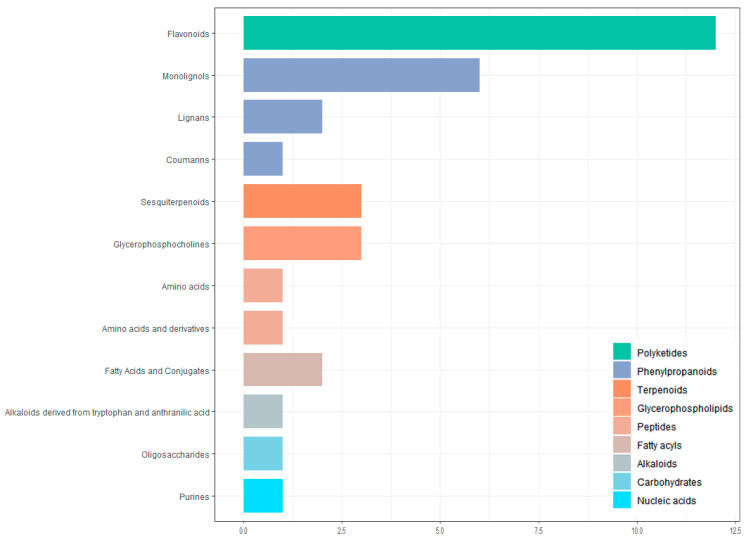
Classification of differential metabolites by KEGG database annotation.

All screened differential metabolites were annotated to the KEGG database (Figure 10). The metabolites were divided into nine categories, including polyketides, phenylpropanoids, terpenoids, glycerophospholipids, peptides, fatty acyls, alkaloids, carbohydrates, and nucleic acids. The largest number of metabolites is flavonoids (12 in total).

**Figure 11:**
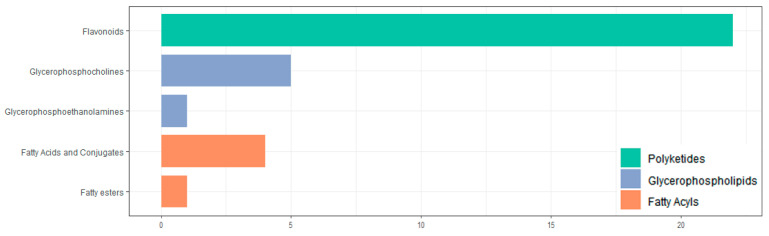
Classification of differential metabolites by LIPID MAPS database annotation.

All screened differential metabolites were annotated to the LIPID MAPS database (Figure 11). The metabolites were divided into three categories, including polyketides, glycerophospholipids, and fatty acyls. The largest number of metabolites is flavonoids (22 in total).

### 3.7. KEGG Enrichment Analysis of Differential Metabolites

**Figure 12:**
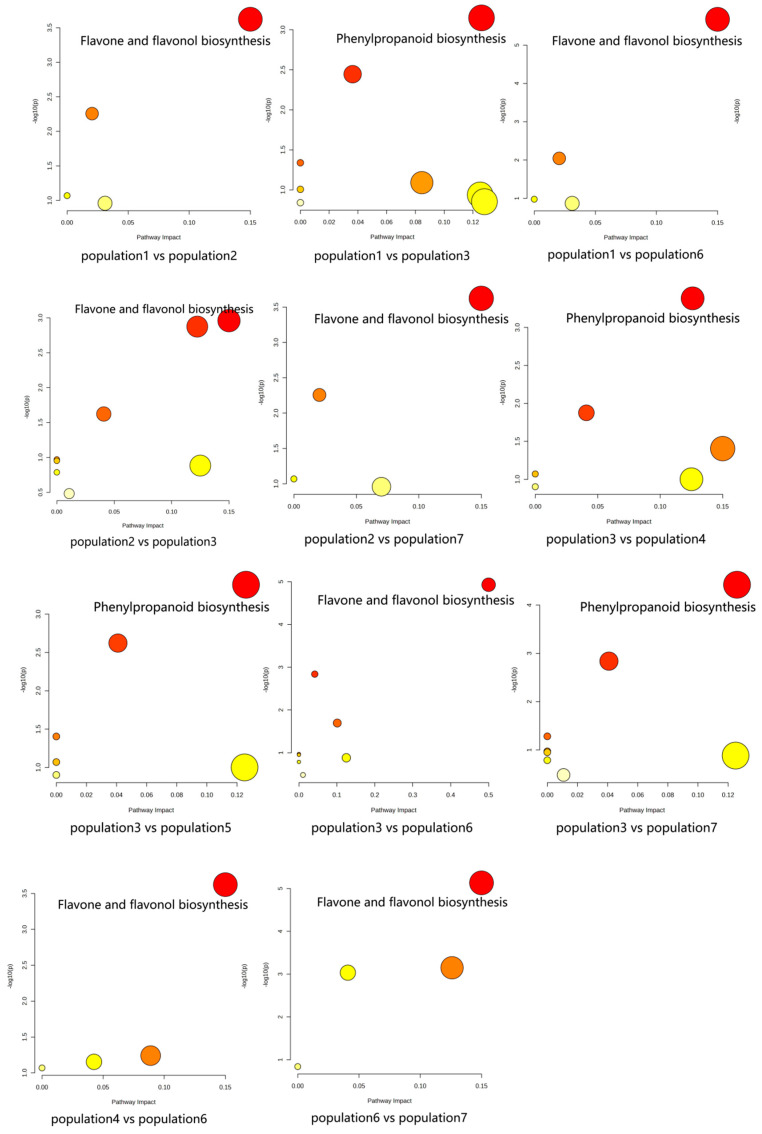

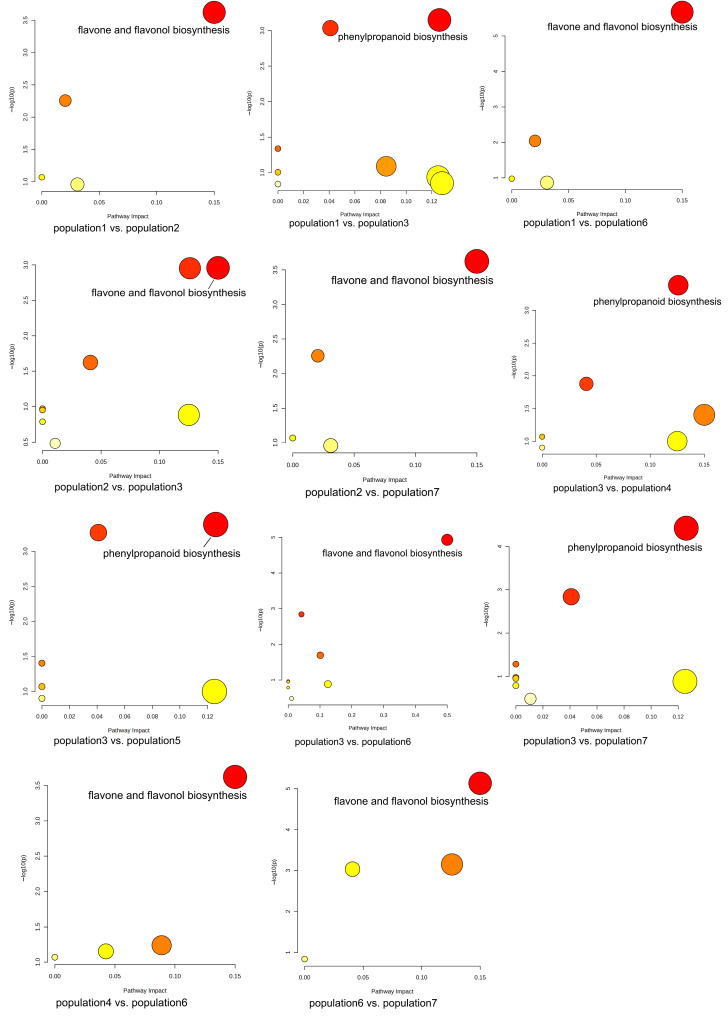
KEGG pathway enrichment of differential metabolites of *S. obv*.

**Figure 13:**
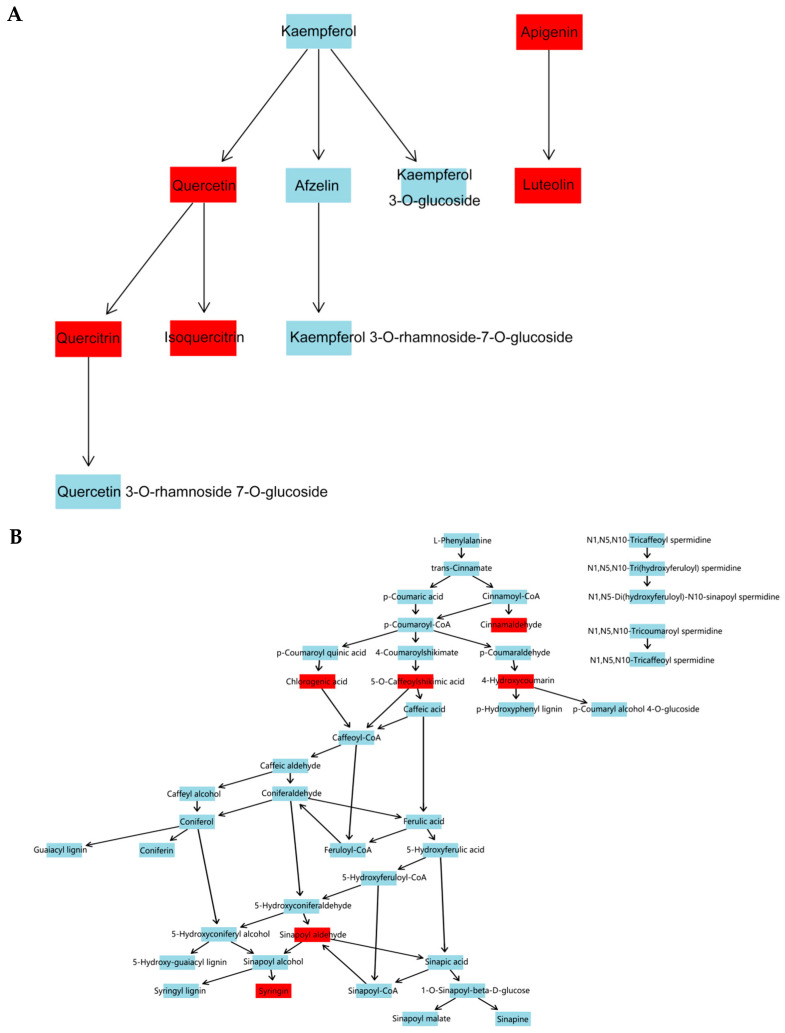
Pathway diagram. (**A**) Flavone and flavonol biosynthesis pathway. (**B**) Phenylpropanoid biosynthesis pathway. Note: The compounds with red label are those identified in *S. obv*.

The results of KEGG pathway-enrichment analysis are shown in Figure 12. The comparison groups with too little annotation information to enrich the pathways or those that did not meet the conditions of significantly enriched pathways were eliminated. In the effective comparison groups, flavone and flavonol biosynthesis and phenylpropanoid biosynthesis were the most significantly enriched pathways. These results indicated that the differential metabolites were mainly related to these two pathways (Figure 13).

### 3.8. Correlation Analysis Between Differential Metabolites and Climatic Factors

**Figure 14:**
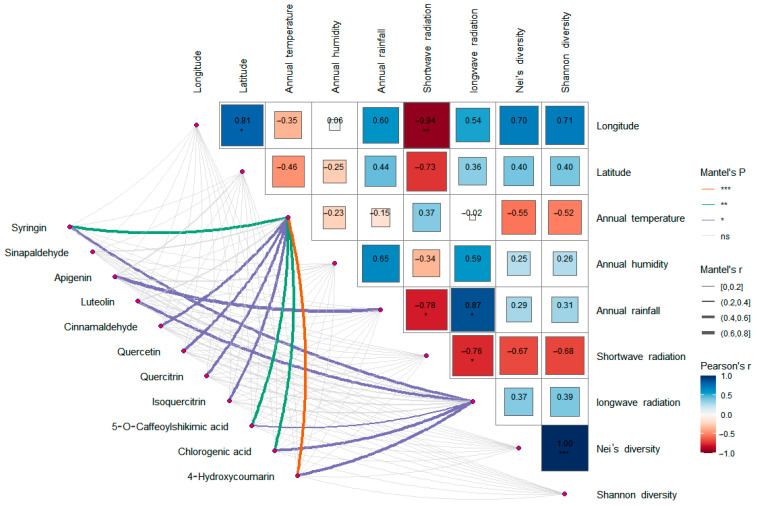
Correlation analysis between differential metabolites, environmental factors, and genetic diversity. * *p* < 0.05, ** *p* < 0.01. *** *p* < 0.001.

A total of 11 differential metabolites associated with the two significantly enriched pathways were screened, including syringin, sinapaldehyde, apigenin, luteolin, cinnamaldehyde, quercetin, quercitrin, isoquercetin, 5-O-caffeoylshikimic acid, chlorogenic acid, and 4-hydroxycoumarin. Correlation analysis (Figure 14) showed that the expression levels of syringin, cinnamaldehyde, quercetin, quercitrin, isoquercetin, 5-O-caffeoylshikimic acid, chlorogenic acid, and 4-hydroxycoumarin were significantly correlated with average annual temperature, apigenin was significantly correlated with average annual rainfall, and syringin, luteolin, 5-O-caffeoylshikimic acid, chlorogenic acid, and 4-hydroxycoumarin were significantly correlated with the annual mean long-wave radiation. There was no significant correlation between the expression levels and genetic diversity.

## 4. Discussion

Genetic diversity is a critical indicator of a species’ ability to survive, reproduce, and evolve. Species with higher genetic diversity would better adapt to environmental changes [47,48,49,50,51]. Wild species of the QTP are threatened by extreme environments, global warming, and inadequate conservation [52,53]. Moreover, the habitats of *S. obv* are relatively moist and densely vegetated, which makes it easy to attract herbivores. Understanding the genetic diversity of *S. obv* is essential for its survival, reproduction, development, and utilization, and it can also play a good role in umbrella protection for other species within the habitats.

Genetic diversity assessments are crucial for designing effective conservation strategies. Nei’s gene diversity (H) among seven populations was 0.3027, and Shannon’s information index (I) was 0.4535. At the population level, these values were lower (H: 0.0834–0.2698; I: 0.1454–0.4145). The genetic differentiation index (Fst) of seven populations was 0.114. Comparatively, studies on *S.* species using different molecular markers revealed divergent patterns. Hu et al. [54] investigated the genetic diversity of *S. involucrata* using simple sequence repeat (SSR) molecular markers, revealing high genetic diversity (H = 0.470 and I = 0.837) among 11 populations in Xinjiang, China. Another genetic diversity analysis of *S. involucrata* using ITS and chloroplast fragments sequences found higher total nucleotide diversity in ITS1-4 region (π = 6.13) than that of cpDNA (π = 1.26), while the total haplotype diversity in the ITS1-4 region (Hd = 0.813) was similar to that of cpDNA (Hd = 0.895) [55]. Twenty *S. medusa* populations in the Qilian Mountains, China, also showed similar genetic diversity (H = 0.2757 and I = 0.4237) yet strong genetic differentiation (Gst = 0.4926), as sequence-related amplified polymorphism (SRAP) markerswere used [56]. The ISSR marker was used to study the genetic diversity of *S. dorogostaiskii*, a globally threatened species, and the results show low genetic diversity (H = 0.17, I = 0.25) but high genetic differentiation (Gst = 0.49) [57]. Semwal et al. [40] studied the genetic diversity of five populations of *S. obv* using an ISSR marker in India, revealing low genetic diversity (H = 0.27 and I = 0.38) due to habitat fragmentation. A broad review [58] comprising 108 entries revealed mean within-population H = 0.214 ± 0.117 and mean Gst = 0.29 ± 0.21. Generally, the genetic diversity and differentiation of *S. obv* were relatively low.

There was no significant correlation between genetic diversity and environmental factors, but this does not necessarily mean that the growth and reproduction of *S. obv* are not restricted by the environment. Instead, it may reflect its adaptation to specific microhabitats. The habitats of different populations are similar, with altitudes between 4000–4500 m, the presence of streams, diurnal temperature variation from June to September, and bushes or boulders to withstand strong winds. Therefore, this species may have strict environmental requirements, which further limit distribution. Moreover, genetic diversity is also associated with life history traits (life form, geographical range, breeding system, seed dispersal, and successional status) and sampling strategies [58]. The genetic diversity of *S. obv* may be shaped by the combined effects of several factors: short-lived perennial life form, endemic to the QTP, mixed breeding system, and seed dispersal by wind. Therefore, further study should incorporate a broader range of sampling sites and life history traits, which would provide valuable insights into the conservation strategies of *S. obv*.

However, the ISSR marker relies on PCR products to analyze genetic diversity. Thus, it cannot obtain information such as gene mutation, and it is difficult to distinguish different bands with very small base number differences. Therefore, the genetic differentiation between different populations remains to be further explored. Chloroplast genome sequencing has been widely applied in phylogenetic studies of the Asteraceae family. According to GenBank (https://www.ncbi.nlm.nih.gov/, accessed on 18 March 2025), the chloroplast genomes of more than 100 *S.* species have been sequenced, and several highly variable regions have been identified [59,60]. This provides new insights into investigating the genetic diversity of *S. obv*.

In this study, UPLC-Q-TOF-MS/MS was used, and 110 components were identified from the ethanol extract of *S. obv*, among which flavonoids and phenylpropanoids were the two most abundant categories.

According to the PCA analysis, there were significant differences in metabolites among different populations. Correlation analysis with environmental factors and genetic diversity showed that the differences in metabolites among populations were not significantly related to geographical distance or genetic diversity. The metabolites of Population 2 and Population 6 were the most similar. In terms of geographical distance, Sample 2 was close to Sample 1, Sample 3, and Sample 4 but far from Sample 6, which further proved that there was no significant correlation between metabolic differences among populations and geographical distance.

Pairwise comparison of metabolites showed that the number of upregulated differential metabolites ranged from 8 to 23, and downregulated differential metabolites ranged from 8 to 26. There was no significant difference in the number of differentiated metabolites between upregulated and downregulated groups, but the magnitude of variation was different. Interestingly, Population 5, which exhibited the lowest genetic diversity, also showed the most pronounced metabolic differentiation compared to other populations. Among all the differential metabolites, the upregulation of arctiin was the highest (Population 1 vs. Population 5, log_2_FC = 11.559), and the downregulation of Curdionolide B was the highest (Population 2 vs. Population 5, log_2_FC = −12.486). Among the environmental factors, Sample 5 had the highest annual mean temperature, lower annual mean precipitation and annual mean moisture content, and stronger short-wave radiation and weaker long-wave radiation, indicating that Sample 5 had greater degrees of high temperature, drought, and temperature fluctuation than other sample sites, which likely contributed to its distinct metabolic profile. In follow-up studies, we should pay more attention to Population 5.

During the growth of plants, a metabolite does not exist independently but interacts with a variety of other metabolites and is regulated by the environment, genes, transcription, and other factors, thus forming a complex metabolic network. Annotation and KEGG pathway enrichment showed that the differential metabolites were mostly flavonoids and related to two pathways: flavone and flavonol biosynthesis and phenylpropanoid biosynthesis. Flavonoids are secondary metabolites commonly foundin plants, and they are also important medicinal substances in medicinal plants, usually with anti-inflammatory, antibacterial, antioxidant, anti-tumor, cardiovascular protection, and other effects [61,62,63,64]. Flavonoids are also important substances for plants to cope with environmental stress, and low temperature and ultraviolet radiation can promote the accumulation of flavonoids [65,66]. Phenylpropanoids are a class of natural products with one or more C6-C3 groups, which have good potential in anti-oxidation, anti-tumor, anti-virus, regulation of blood sugar, and protection of ultraviolet radiation damage [67,68]. Phenylpropanoids can enhance plant resistance to insect pests, cold resistance, oxidation resistance, and mechanical damage [69,70]. Some volatile phenylpropanoids, such as benzyl alcohol, benzyl acetate, methyl benzoate, and phenylacetaldehyde, can attract pollinating insects and play an important role in plant reproduction [71]. The biosynthesis of phenylpropanoids is regulated by biological and abiotic factors. Studies have shown that low temperature can promote the accumulation of lignin and anthocyanins in the phenylpropanoids biosynthesis pathway [72,73,74], and lignin can enhance the toughness of the plant cell wall to cope with the mechanical damage caused by low temperature. Anthocyanins have good antioxidant capacity, which can reduce ROS accumulation and alleviate oxidative damage caused by low-temperature stress on plants. In addition, syringin and other phenylpropanoids were also affected by drought stress [75]. Metal ion concentration (including copper, iron, zinc, manganese, and sodium) [76] and salt stress [77] can also affect the pathways related to flavonoids and phenylpropanoids and then promote or inhibit the expression of flavonoids and phenylpropanoids.

Among the differential metabolites of different populations, 11 differential metabolites related to these two pathways were screened out, including syringin, sinapaldehyde, apigenin, luteolin, cinnamaldehyde, quercetin, quercitrin, isoquercetin, 5-O-caffeoylshikimic acid, chlorogenic acid, and 4-hydroxycoumarin. Correlation analysis of these 11 differential metabolites with environmental factors and genetic diversity showed that there was no significant correlation between the expression levels of these 11 differential metabolites and genetic diversity, latitude, and longitude, indicating that the metabolites of *S. obv* were mainly affected by environmental factors rather than genetic factors. Also, there is no obvious spatial structure on a large scale. Among the environmental factors, the annual mean temperature had the greatest influence on the differential metabolites. There were eight differential metabolites (syringin, cinnamaldehyde, quercetin, quercetin, isoquercetin, 5-O-cafeoylshikimic acid, chlorogenic acid, and 4-hydroxycoumarin) that were significantly correlated with the annual mean temperature, followed by the annual mean long-wave radiation. Five different metabolites (syringin, luteolin, 5-O-caffeoylshikimic acid, chlorogenic acid, and 4-hydroxycoumarin) were significantly correlated with the annual mean long-wave radiation amount, suggesting that the different metabolites were mainly related to temperature and diurnal temperature variation. The responses of plant secondary metabolite accumulation to temperature have been proven [78], while the mechanisms, which are how temperature and diurnal temperature variation affect the above metabolites, remain to be studied.

Although the mean annual precipitation and mean annual moisture content of each sample site were significantly different, there was almost no significant correlation between the two factors and the expression levels of different metabolites, except for apigenin. This may be due to the fact that *S. obv* grows near waterfalls or streams. Even when precipitation is low, the water flow can also make up for the lack of precipitation. *S. obv* mainly grows in summer and autumn. During this period, the QTP has higher temperature and precipitation, leading to abundant river water, so *S. obv* may not be susceptible to drought stress.

## 5. Conclusions

In conclusion, this study provides valuable insights into the genetic and metabolic diversity of *S. obv*, emphasizing the importance of environmental factors in shaping its adaptive responses. The low genetic diversity and limited gene flow among populations underscore the need for conservation strategies. Furthermore, the identification of key metabolites and their associated pathways offers a foundation for future research into the pharmacological potential of *S. obv*. By integrating genetic and metabolomic approaches, this study contributes to a deeper understanding of the ecological and medicinal significance of this endangered species, paving the way for its sustainable utilization and conservation.

## Figures and Tables

**Table 1:** Geographical characteristics of seven populations of *S. obv*.

Site No.	Location	Longitude (E)	Latitude (N)	Altitude (m)	Specimen No.
1	Duoda village, Xiayadong town, Yadong county, Xigaze City, Xizang	89°04′21″	28°02′21″	4150	542334220721001
2	Zhuola mountain, Xiayadong town, Yadong county, Xigaze City, Xizang	88°48′55″	27°26′09″	4300	542334220722001
3	Shangyadong town, Yadong county, Xigaze City, Xizang	89°38′01″	28°15′46″	4020	542334220722004
4	Kangbu town, Yadong county, Xigaze City, Xizang	88°58′44″	27°51′23″	4210	542334220723001
5	Chentang town, Dingjie county, Xigaze City, Xizang	87°42′02″	28°26′06″	4050	540231220724001
6	Sejila Mountain, Bayi District, Nyingchi City, Xizang	95°05′09″	30°01′58″	4598	540402220727001
7	Lebugou, Cuona county, Shannan City, Xizang	91°51′10″	27°55′15″	4020	540530220807001

**Table 2:** Annealing temperature, bands, and polymorphism.

Primer	Best Annealing Temperature (°C)	Bands	Polymorphic Bands
808	52	10	10
809	52	7	7
811	52	10	10
812	52	9	9
814	48	9	9
817	48	10	10
818	52	9	9
819	52	8	8
829	52	9	9
842	48	8	8
850	48	6	6
851	48	8	8
852	48	9	9
862	48	6	6
865	48	7	7
867	48	9	9
884	48	11	11
886	48	15	15
889	48	15	15
890	48	10	10

**Table 3:** Intra-population genetic diversity of seven populations of *S. obv*.

Population	Np	PPL	Na	Ne	H	I
1	60	58.25%	1.5825	1.2478	0.1607	0.2549
2	64	62.14%	1.6214	1.2243	0.1505	0.2443
3	78	75.73%	1.7573	1.3909	0.2375	0.3641
4	82	79.61%	1.7961	1.3976	0.2436	0.3751
5	47	45.63%	1.4563	1.1122	0.0834	0.1454
6	89	86.41%	1.8641	1.4439	0.2698	0.4145
7	93	90.29%	1.9029	1.3834	0.2434	0.3852
Average	73.29	71.15%	1.7115	1.3143	0.1984	0.3119

Np: No. of polymorphic loci; PPL: percentage of polymorphic loci; Na: No. of observed alleles; Ne: No. of effective alleles; H: Nei’s gene diversity; I: Shannon’s information index.

**Table 4:** AMOVA analysis of seven populations of *S. obv*.

Source of Differentiation	df	SS	MS	Est. Var	Proportion	Fst	*p*
Inter-population	6	152.050	25.342	0.913	11%	0.114	0.001
Within population	133	940.850	7.074	7.074	89%		
Total	139	1092.900		7.987	100%		

df: degree of freedom; SS: sum of standard deviation; MS: mean of standard deviation; Est. Var: estimated variance; Fst: genetic differentiation index.

**Table 5:** Genetic identity and genetic distance among seven populations of *S. obv*.

Population	1	2	3	4	5	6	7
1	-	0.3216	0.5680	0.3567	0.3687	0.7802	0.3808
2	0.7250	-	0.3216	0.4437	0.2025	0.5680	0.3216
3	0.5667	0.7250	-	0.2657	0.3687	0.5534	0.5978
4	0.7000	0.6417	0.7667	-	0.3930	0.5828	0.5680
5	0.6917	0.8167	0.6917	0.6750	-	0.5108	0.3448
6	0.4583	0.5667	0.5750	0.5583	0.6000	-	0.5248
7	0.6833	0.7250	0.5500	0.5667	0.7083	0.5917	-

Note: Below diagonal is genetic identity, and above diagonal is genetic distance.

**Table 6:** Climatic factors of seven sampling sites.

Site No.	Temperature (°C)	Moisture Content (‰)	Rainfall (mm)	Short-Wave Radiation (W/m^2^)	Long-Wave Radiation (W/m^2^)
1	0.79	4.94	421.58	219.48	247.21
2	0.17	8.04	799.36	210.51	275.18
3	−2.04	5.60	445.67	224.91	232.37
4	0.37	6.29	572.15	216.67	273.95
5	2.01	4.21	504.26	236.87	244.89
6	−1.43	5.74	830.57	174.22	283.36
7	1.98	5.93	648.25	204.3	263.56

## Data Availability

The original contributions presented in this study are included in the article/Supplementary Material. Further inquiries can be directed at the corresponding author.

## Supplementary Materials

The following supporting information can be downloaded at: https://www.mdpi.com/article/10.3390/genes16050593/s1, Table S1: Sequences of 100 ISSR universal primers; Table S2: PCR amplification procedures; Table S3: The eluent gradient; Table S4: Mass parameters; Table S5: Compounds identified from *S. obv* ethanol extract by UPLC-Q-TOF-MS/MS; Table S6: List of the 85 differential metabolites.

## Abbreviations

The following abbreviations are used in this manuscript:

AMOVA	Analysis of molecular variance
AFLP	Amplified fragment length polymorphism
CAMP	Conservation Assessment Management Plan
CITES	Convention of International Trade in Endangered Species of Wild Fauna and Flora
df	Degree of freedom
Est. Var	Estimated variance
Fst	Genetic differentiation index
H	Nei’s gene diversity
I	Shannon’s information index
ISSR	Inter-simple sequence repeat
MS	Mean of standard deviation
Na	Number of observed alleles
Ne	Number of effective alleles
Np	Number of polymorphic loci
PCA	Principal component analysis
PCR	Polymerase chain reaction
PPL	Percentage of polymorphic bands
QC	Quality control
QTP	Qinghai-Tibet Plateau
SRAP	Sequence-related amplified polymorphism
SS	Sum of standard deviation
SSR	Simple sequence repeat
UPLC-Q-TOF-MS/MS	Ultra-high-performance liquid chromatography-tandem quadrupole-time-of-flight mass spectrometry
