# Supplementary material for: Genetic Diversity and Metabolic Profile of Tibetan Medicinal Plant Saussurea obvallata
Source: Genes (Basel). 2025 May 17;16(5):593. doi: 10.3390/genes16050593 (PMC12110891; doi:10.3390/genes16050593)
Supplement: Supplementary file 1 [file genes-16-00593-s001.zip › genes-3593319-supplementary.pdf]

Table S1 Sequences of 100 ISSR universal primer

Primer	Sequence (5'to3')
801	ATA TAT ATA TAT ATA TT
802	ATA TAT ATA TAT ATA TG
803	ATA TAT ATA TAT ATA TC
804	TAT ATA TAT ATA TAT AA
805	TAT ATA TAT ATA TAT AC
806	TAT ATA TAT ATA TAT AG
807	AGA GAG AGA GAG AGA GT
808	AGA GAG AGA GAG AGA GC
809	AGA GAG AGA GAG AGA GG
810	GAG AGA GAG AGA GAG AT
811	GAG AGA GAG AGA GAG AC
812	GAG AGA GAG AGA GAG AA
813	CTC TCT CTC TCT CTC TT
814	CTC TCT CTC TCT CTC TA
815	CTC TCT CTC TCT CTC TG
816	CAC ACA CAC ACA CAC AT
817	CAC ACA CAC ACA CAC AA
818	CAC ACA CAC ACA CAC AG
819	GTG TGT GTG TGT GTG TA
820	GTG TGT GTG TGT GTG TC
821	GTG TGT GTG TGT GTG TT
822	TCT CTC TCT CTC TCT CA
823	TCT CTC TCT CTC TCT CC
824	TCT CTC TCT CTC TCT CG
825	ACA CAC ACA CAC ACA CT
826	ACA CAC ACA CAC ACA CC
827	ACA CAC ACA CAC ACA CG
828	TGT GTG TGT GTG TGT GA
829	TGT GTG TGT GTG TGT GC
830	TGT GTG TGT GTG TGT GG
831	ATA TAT ATA TAT ATA TYA
832	ATA TAT ATA TAT ATA TYC
833	ATA TAT ATA TAT ATA TYG
834	AGA GAG AGA GAG AGA GYT
835	AGA GAG AGA GAG AGA GYC
836	AGA GAG AGA GAG AGA GYA
837	TAT ATA TAT ATA TAT ART
838	TAT ATA TAT ATA TAT ARC
839	TAT ATA TAT ATA TAT ARG

840	GAG AGA GAG AGA GAG AYT
841	GAG AGA GAG AGA GAG AYC
842	GAG AGA GAG AGA GAG AYG
843	CTC TCT CTC TCT CTC TRA
844	CTC TCT CTC TCT CTC TRC
845	CTC TCT CTC TCT CTC TRG
846	CAC ACA CAC ACA CAC ART
847	CAC ACA CAC ACA CAC ARC
848	CAC ACA CAC ACA CAC ARG
849	GTG TGT GTG TGT GTG TYA
850	GTG TGT GTG TGT GTG TYC
851	GTG TGT GTG TGT GTG TYG
852	TCT CTC TCT CTC TCT CRA
853	TCT CTC TCT CTC TCT CRT
854	TCT CTC TCT CTC TCT CRG
855	ACA CAC ACA CAC ACA CYT
856	ACA CAC ACA CAC ACA CYA
857	ACA CAC ACA CAC ACA CYG
858	TGT GTG TGT GTG TGT GRT
859	TGT GTG TGT GTG TGT GRC
860	TGT GTG TGT GTG TGT GRA
861	ACC ACC ACC ACC ACC ACC
862	AGC AGC AGC AGC AGC AGC
863	AGT AGT AGT AGT AGT AGT
864	ATG ATG ATG ATG ATG ATG
865	CCG CCG CCG CCG CCG CCG
866	CTC CTC CTC CTC CTC CTC
867	GGC GGC GGC GGC GGC GGC
868	GAA GAA GAA GAA GAA GAA
869	GTT GTT GTT GTT GTT GTT
870	TGC TGC TGC TGC TGC TGC
871	TAT TAT TAT TAT TAT TAT
872	GAT AGA TAG ATA GAT A
873	GAC AGA CAG ACA GAC A
874	CCC TCC CTC CCT CCC T
875	CTA GCT AGC TAG CTA G
876	GAT AGA TAG ACA GAC A
877	TGC ATG CAT GCA TGC A
878	GGA TGG ATG GAT GGA T
879	CTT CAC TTC ACT TCA
880	GGA GAG GAG AGG AGA
881	GGG TGG GGT GGG GTG

882	VBV ATA TAT ATA TAT AT
883	BVB TAT ATA TAT ATA TA
884	HBH AGA GAG AGA GAG AG
885	BHB GAG AGA GAG AGA GA
886	VDV CTC TCT CTC TCT CT
887	DVD TCT CTC TCT CTC TC
888	BDB CAC ACA CAC ACA CA
889	DBD ACA CAC ACA CAC AC
890	VHV GTG TGT GTG TGT GT
891	HVH TGT GTG TGT GTG TG
892	TAG ATC TGA TAT CTG AAT TCC C
893	NNN NNN NNN NNN NNN
894	TGG TAG CTC TTG ATC ANN NNN
895	AGA GTT GGT AGC TCT TGA TC
896	AGG TCG CGG CCG CNN NNN NAT G
897	CCG ACT CGA GNN NNN NAT GTG G
898	GAT CAA GCT TNN NNN NAT GTG G
899	CAT GGT GTT GGT CAT TGT TCC A
900	ACT TCC CCA CAG GTT AAC ACA

N= (A, G, C, T), R= (A, G), Y= (C, T), B= (C, G, T), D= (A, G, T)

Table S2 PCR amplification procedures

Procedure	Temperature(℃)	Time	Cycles
Initial denaturation	94	3min	1
Denaturation	94	30s	
Annealing	45/48/52	30s	35
Extension	72	1min	
Extension	72	5min	1

Table S3 The eluent gradient

Time (min)	Percentage of A (%)
0	5
3	10
8	20
10	20
15	30
20	65
25	95
26	5
30	5

Table S4 Mass parameters

MS parameter	Value	MS/MS parameter	Value
TOF mass range (*m/z*)	100-2000	MS/MS mass range (*m/z*)	100-2000
Ion Source Gas 1 (psi)	20	Declustering Potential (V)	80
Ion Source Gas 2 (psi)	0	DP spread (V)	0
Curtain Gas (psi)	25	Collision Energy (eV)	35
Temperature (℃)	0	CE spread (eV)	15
Ion Spray Voltage Floating (V)	-4500/5500	Accumulation time	0.05
Declustering Potential (V)	80	Maximum candidate ions	10
Collision Energy (eV)	10	Intensity threshold exceeds	100
Accumulation time	0.25		

Table S5 Compounds identified from *S. obvallata* ethanol extract by UPLC-Q-TOF-MS/MS

No.	Retention time (min)	Adduct ion	Formula	*m/z* theoretical	*m/z* calculated	Mass Error (ppm)	Identification	Classification
1	1.04	[M+H]+	C6H14N4O2	175.1195	175.1190	-2.86	L(+)-Arginine	amino acids and derivatives
2	1.05	[M+H]+	C5H13NO	104.1075	104.1071	-4.22	Choline	alkaloids
3	1.08	[M+H]+	C5H11NO2	118.0868	118.0863	-4.27	Betaine	amino acids and derivatives
4	1.09	[M+H]+	C5H9NO2	116.0712	116.0714	2.12	Proline	amino acids and derivatives
5	1.1	[M+H]+	C4H7N	70.0657	70.0656	-1.06	1-Pyrroline	azole
6	1.1	[M+H]+	C7H7NO2	138.0555	138.0551	-2.93	Anthranilic acid	amino acids and derivatives
7	1.13	[M+H]+	C7H7NO2	138.0555	138.0551	-2.93	Trigonelline	alkaloids
8	1.13	[M+H]+	C11H17NO6	260.1134	260.1130	-1.59	O-Fumarylcarnitine	nitrogenous organic compounds
9	1.154	[M+H]+	C16H16O8	337.0923	337.0918	-1.62	5-O-Caffeoylshikimic acid	phenylpropanoids
10	1.17	[M+H]+	C8H9NO	136.0762	136.0760	-1.76	2-Phenylacetamide	acetamides
11	1.18	[M+H]+	C5H5N5	80.0500	80.0501	0.95	Adenine	nucleic acids
12	1.18	[M+H]+	C15H19NO6	310.1291	310.1291	0.12	Esculamine	glycosides
13	6.73	[M+H]+	C9H8O	133.0653	133.0654	0.45	Cinnamaldehyde	aldehydes
14	6.75	[M+H]+	C11H12O3	193.0865	193.0860	-2.43	Methyl 4-methoxycinnamate	carboxylic acids
15	6.75	[M+H]+	C17H24O9.NH3	390.1764	390.1764	-0.02	Syringin	phenylpropanoids
16	7.24	[M+H]+	C16H18O9	355.1029	355.1020	-2.56	Chlorogenic acid	phenylpropanoids
17	8.59	[M+H]+	C33H40O21	773.2140	773.2132	-1.09	Quercetin-3-O-rutinosyl-7-O-glucoside	flavonoids
18	8.65	[M+H]+	C11H12O4	209.0814	209.0811	-1.36	Sinapaldehyde	phenylpropanoids
19	11.583	[M-H2O+H]+	C26H32O11	503.1917	503.1909	-1.64	2-(hydroxymethyl)-6-[4-[(2S,3S)-3-(hydroxymethyl)-5-[(E)-3-hydroxyprop-1-enyl]-7-methoxy-2,3-dihydro-1-benzofuran-2-yl]-2-methoxyphenoxy]oxane-3,4,5-triol	-
20	12.148	[M+H]+	C27H30O15	595.1663	595.1658	-0.84	Luteolin 7-rutinoside	flavonoids

21	12.15	[M+H]+	C15H10O7	303.0505	303.0500	-1.58	5,7-dihydroxy-2-(2,3,4-trihydroxyphenyl)chromen-4-one	-
22	12.15	[M+H]+	C21H20O11	449.1084	449.1082	-0.42	Quercitrin	flavonoids
23	12.15	[M+H]+	C21H20O12	465.1033	465.1030	-0.66	Hyperin	flavonoids
24	12.16	[M+H]+	C27H30O16	611.1612	611.1613	0.14	Rutin	flavonoids
25	12.188	[M+H]+	C15H10O7	303.0505	303.0495	-3.23	Bracteatin	flavonoids
26	12.188	[M+H]+	C21H20O12	465.1033	465.1030	-0.66	Isoquercitrin	flavonoids
27	12.2	[M+H]+	C15H10O7	303.0505	303.0500	-1.58	Quercetin	flavonoids
28	13.86	[M+H]+	C15H10O6	287.0556	287.0551	-1.62	Kaempferol	flavonoids
29	13.87	[M+H]+	C15H10O6	287.0556	287.0551	-1.62	Luteolin	flavonoids
30	13.903	[M+H]+	C27H30O15	595.1663	595.1653	-1.68	Kaempferol-3-O-glucosyl(1-2)rhamnoside	flavonoids
31	13.93	[M+H]+	C8H6O2	135.0446	135.0440	-4.48	Phthalide	heterocyclic compounds
32	13.969	[M-H2O+H]+	C25H24O12	499.1240	499.1233	-1.48	Isochlorogenic acid A	phenylpropanoids
33	13.98	[M+H]+	C9H6O3	163.0395	163.0392	-1.96	4-Hydroxycoumarin	phenylpropanoids
34	15.294	[M+H]+	C21H18O13	479.0826	479.0817	-1.82	Querciturone	flavonoids
35	16	[M+H]+	C21H18O12	463.0877	463.0870	-1.41	Luteolin 7-glucuronide	flavonoids
36	16.545	[M+H]+	C21H20O11	449.1084	449.1079	-1.09	Luteolin4'-o-glucoside	flavonoids
37	18.02	[M+H]+	C21H24O6	373.1651	373.1651	-0.04	Arctigenin	phenylpropanoids
38	18.02	[M+H]+	C27H34O11.NH3	552.2445	552.2442	-0.52	Arctiin	phenylpropanoids
39	18.55	[M+H]+	C25H24O14	549.1244	549.1240	-0.79	Geranitin O-malonyl glucoside	-
40	18.88	[M+H]+	C20H27NO4	346.2018	346.2010	-2.41	Saussureamines B	terpenoids
41	19.46	[M+H]+	C28H32O14	593.1870	593.1862	-1.41	Linarin	flavonoids
42	19.508	[M+H]+	C28H32O14	593.1870	593.1866	-0.73	5-hydroxy-3-(4-methoxyphenyl)-7-[3,4,5-trihydroxy-6-[(3,4,5-trihydroxy-6-methyloxan-2-yl)oxymethyl]oxan-2-yl]oxychromen-4-one	-
43	19.68	[M+H]+	C15H16O2	229.1229	229.1220	-3.73	Gweicurculactone	terpenoids
44	20.35	[M+H]+	C22H23NO4	366.1705	366.1700	-1.46	Dehydrocorydaline	alkaloids

45	20.9	[M+H]+	C15H20O3	249.1491	249.1491	0.12	Curdionolide B	heterocyclic compounds
46	20.91	[M+H]+	C15H20O3	249.1491	249.1491	0.12	Parthenolide	terpenoids
47	21.1	[M+H]+	C9H10O	135.0810	135.0812	1.55	2-Phenylpropionaldehyde	aldehydes
48	21.16	[M+H]+	C9H10O	135.0810	135.0812	1.55	3,4-Dimethylzaldehyde	aldehydes
49	22.019	[M-H2O+H]+	C15H22O3	233.1542	233.1532	-4.10	2-Naphthaleneacetic acid, decahydro-4-hydroxy-4a-methyl-alpha,8-bis(methylene)-	-
50	22.41	[M+H]+	C15H10O5	271.0607	271.0600	-2.40	Apigenin	flavonoids
51	22.54	[M+H]+	C16H12O6	301.0712	301.0710	-0.71	Tectorigenin	flavonoids
52	22.56	[M+H]+	C16H12O6	301.0712	301.0707	-1.71	Scutellarein 4'-methyl ether	flavonoids
53	22.62	[M+H]+	C25H31N3O4	438.2393	438.2390	-0.64	Dicoumaroyl Spermidine	alkaloids
54	23.11	[M+H]+	C8H8O2	137.0603	137.0600	-1.86	Anisaldehyde	aldehydes
55	23.41	[M+H]+	C9H6O2	147.0446	147.0440	-4.11	Coumarin	phenylpropanoids
56	23.422	[M+H]+	C46H50N4O8	787.3707	787.3703	-0.50	N1,N5,N10,N14-Tetra-trans-p-coumaroylspermine	carboxylic acids
57	23.55	[M+H]+	C15H18O	215.1436	215.1430	-2.74	8-Hydroxycadalene	terpenoids
58	23.55	[M+H]+	C15H20O2	233.1542	233.1540	-0.66	Isoalantolactone	terpenoids
59	23.55	[M+H]+	C15H20O2	233.1542	233.1540	-0.66	Costunolide	terpenoids
60	24.19	[M+H]+	C18H26O3	291.1960	291.1950	-3.50	Octinoxate	carboxylic acids
61	24.53	[M+H]+	C16H35NO2	274.2746	274.2740	-2.20	N-Lauryldiethanolamine	fatty acids
62	24.62	[M+H]+	C14H31NO	230.2484	230.2480	-1.69	Lauramine oxide	fatty acids
63	24.63	[M+H]+	C18H39NO3	318.3008	318.3000	-2.57	Phytosphingosine	lipids
64	24.7	[M+H]+	C16H12O5	285.0763	285.0760	-1.05	Acacetin	flavonoids
65	24.84	[M+H]+	C17H14O6	315.0869	315.0860	-2.75	Pectolinarigenin	flavonoids
66	25.37	[M+H]+	C24H30O6	415.2121	415.2120	-0.16	HX-3 1	-
67	25.71	[M+H]+	C26H48NO7P	518.3247	518.3240	-1.29	LPC 18:3	lipids
68	25.98	[M+H]+	C15H16O	213.1279	213.1270	-4.41	Pyrocurzerenone	lipids

69	25.98	[M+H]+	C15H18O2	231.1385	231.1380	-2.18	Dehydrocostus lactone	terpenoids
70	25.99	[M-H2O+H]+	C15H20O3	231.1385	231.1374	-4.78	Reynosin	lipids
71	26.2	[M+H]+	C18H37NO	284.2953	284.2950	-1.19	Stearamide	lipids
72	26.21	[M+H]+	C18H28O2	277.2168	277.2160	-2.72	Stearidonic acid	lipids
73	26.25	[M+H]+	C23H44NO7P	478.2934	478.2930	-0.77	LPE 18:2	lipids
74	26.27	[M+H]+	C26H50NO7P	520.3403	520.3400	-0.61	LPC 18:2	lipids
75	26.48	[M+H]+	C19H38O4	331.2848	331.2840	-2.52	Jaceosidin	flavonoids
76	26.618	[M-H2O+H]+	C18H28O3	275.2011	275.2003	-2.93	8-{(1S,5R)-4-Oxo-5-[(2Z)-2-penten-1-yl]-2-cyclopenten-1-yl}octanoic acid	-
77	26.63	[M+H]+	C21H44NO7P	454.2934	454.2930	-0.81	LPE 16:0	lipids
78	26.69	[M+H]+	C24H50NO7P	496.3403	496.3400	-0.64	LPC 16:0	lipids
79	26.7	[M+H]+	C15H22O2	235.1698	235.1690	-3.42	3,5-di-tert-butyl-4-hydroxybenzaldehyde	aldehydes
80	26.89	[M+H]+	C18H39O7P	399.2512	399.2510	-0.42	Tributoxyethyl phosphate	phosphorous organic compounds
81	26.95	[M+H]+	C26H52NO7P	522.3560	522.3550	-1.85	LPC 18:1	lipids
82	27.044	[M+NH4]+	C9H9NO2	181.0977	181.0971	-3.33	Resedine	phenylpropanoids
83	27.3	[M+H]+	C8H4O3	149.0239	149.0232	-4.50	Phthalic anhydride	phenylpropanoids
84	27.73	[M+H]+	C21H36O4	353.2692	353.2690	-0.52	Monolinolenin (9c,12c,15c)	fatty acids
85	27.75	[M+H]+	C20H37NO2	324.2903	324.2900	-0.78	Linoleoyl ethanolamide	fatty acids
86	27.78	[M+H]+	C26H54NO7P	524.3716	524.3710	-1.18	LPC 18:0	lipids
87	27.88	[M+H]+	C20H34O8	403.2332	403.2330	-0.48	Acetyltributyl citrate	carboxylic acids
88	27.91	[M+H]+	C6H4O5	157.0137	157.0130	-4.46	2,5-Furandicarboxylic acid	heterocyclic compounds
89	28.29	[M+H]+	C18H30O2	279.2324	279.2320	-1.45	Linolenic acid	fatty acids
90	28.61	[M+H]+	C16H33NO	256.2640	256.2630	-4.05	Palmitamide	fatty acids
91	28.8	[M+H]+	C18H35NO	282.2797	282.2790	-2.44	Oleamide	fatty acids
92	28.81	[M+H]+	C19H36O3	313.2743	313.2740	-0.86	Methyl 5-oxooctadecanoate	fatty acids

93	28.99	[M+H]+	C42H63O4P	663.4542	663.4540	-0.34	Tris(2,4-di-tert-butylphenyl) phosphate	phosphorous organic compounds
94	29.43	[M+H]+	C22H43NO	338.3423	338.3420	-0.85	Erucamide isomer	fatty acids
95	29.45	[M+H]+	C22H43NO	338.3423	338.3420	-0.85	Erucamide	fatty acids
96	29.5	[M+H]+	C35H36N4O5	593.2764	593.2760	-0.67	Pheophorbide A	heterocyclic compounds
97	29.72	[M+H]+	C33H40N2O9	609.2812	609.2810	-0.34	Reserpine	alkaloids
98	1.11	[M-H]-	C6H6O2	109.0290	109.0291	1.51	Hydroquinone	phenols
99	1.114	[M-H]-	C12H22O11	341.1084	341.1089	1.55	Sucrose	carbohydrates
100	8.621	[M-H]-	C33H40O21	771.1984	771.2020	4.71	3-[(2S,3R,4S,5R,6S)-3,4-dihydroxy-6-methyl-5-[(2S,3R,4S,5S,6R)-3,4,5-trihydroxy-6-(hydroxymethyl)oxan-2-yl]oxyoxan-2-yl]oxy-2-(3,4-dihydroxyphenyl)-5-hydroxy-7-[3,4,5-trihydroxy-6-(hydroxymethyl)oxan-2-yl]oxychromen-4-one	-
101	12.537	[M-H]-	C21H20O11	447.0927	447.0938	2.30	Cynaroside	flavonoids
102	13.115	[M-H]-	C28H36O13	579.2078	579.2094	2.80	Acanthoside B	phenylpropanoids
103	13.754	[M-H]-	C23H22O13	505.0982	505.0994	2.42	Quercetin-3-O-glucose-6''-acetate	flavonoids
104	13.995	[M-H]-	C16H18O9	353.0873	353.0878	1.64	Caffeoylquinic acid	phenylpropanoids
105	14.186	[M-H]-	C25H24O12	515.1190	515.1208	3.54	1,3-Dicaffeoylquinic acid	phenylpropanoids
106	14.453	[M-H]-	C28H32O16	623.1612	623.1632	3.19	Isorhamnetin 3-robinobioside	flavonoids
107	15.702	[M-H]-	C16H18O9	353.0873	353.0888	4.25	(3R,5S)-4-[(E)-3-(3,4-dihydroxyphenyl)prop-2-enoyl]oxy-1,3,5-trihydroxycyclohexane-1-carboxylic acid	-
108	21.176	[M-H]-	C34H30O15	677.1507	677.1538	4.70	3,4,5-Tricaffeoylquinic acid	phenylpropanoids
109	24.882	[M-H]-	C17H14O6	313.0712	313.0719	2.22	Luteolin 3',4'-dimethyl ether	flavonoids
110	26.396	[M+HCOO]-	C27H46O9	559.3118	559.3138	3.58	9,12,15-Octadecatrienoic acid, 3-(hexopyranosyloxy)-2-hydroxypropyl ester, (9Z,12Z,15Z)-	-

Table S6 List of the 85 differential metabolites

No.	Differential metabolites	Comparable groups with significant difference																
1	Arctiin	1v2	1v3	1v4	1v5	1v6	1v7	2v3	2v5	2v6	3v4	3v6	3v7	4v5	4v7	5v6	5v7	6v7
2	Curdionolide B	1v2	1v4	1v5	1v7	2v3	2v4	2v5	2v6	2v7	3v4	3v5	3v7	4v5	4v6	4v7	5v6	6v7
3	Quercituron	1v2	1v3	1v5	1v6	2v4	2v5	2v6	2v7	3v4	3v5	3v6	3v7	4v5	4v6	4v7	5v6	5v7
4	Resedine	1v2	1v3	1v4	1v6	1v7	2v3	2v4	2v5	2v7	3v4	3v5	3v6	4v5	4v6	4v7	5v7	6v7
5	8-Hydroxycadalene	1v2	1v3	1v4	1v6	1v7	2v3	2v4	2v5	2v7	3v5	3v6	4v5	4v6	5v6	5v7	6v7	
6	Arctigenin	1v2	1v3	1v4	1v5	1v6	1v7	2v3	2v5	3v4	3v6	3v7	4v5	4v7	5v6	5v7	6v7	
7	Betaine	1v2	1v3	1v5	1v6	1v7	2v3	2v4	2v7	3v4	3v5	3v6	3v7	4v5	4v6	5v7	6v7	
8	Dehydrocostus lactone	1v4	1v5	1v7	2v4	2v5	2v6	2v7	3v4	3v5	3v7	4v5	4v6	4v7	5v6	6v7		
9	Isoalantolactone	1v2	1v3	1v4	1v5	1v6	2v4	2v5	2v7	3v5	3v6	4v5	4v6	5v6	5v7	6v7		
10	Cynaroside	1v2	1v4	1v6	1v7	2v3	2v4	2v5	2v7	3v7	4v6	4v7	5v6	5v7	6v7			
11	Gweicurculactone	1v2	1v5	1v7	2v4	2v5	2v6	2v7	3v5	3v7	4v5	4v6	4v7	5v6	6v7			
12	Pyrocurzerenone	1v4	1v5	1v7	2v4	2v5	2v6	2v7	3v5	3v7	4v5	4v6	4v7	5v6	6v7			
13	Reynosin	1v4	1v5	1v7	2v4	2v5	2v6	2v7	3v5	3v7	4v5	4v6	4v7	5v6	6v7			
14	Saussureamines B	1v4	1v5	1v7	2v4	2v5	2v7	3v4	3v5	3v7	4v5	4v7	5v6	5v7	6v7			
15	Scutellarein 4'-methyl ether	1v2	1v3	1v4	1v6	2v3	2v4	2v5	2v7	3v6	4v6	4v7	5v6	5v7	6v7			
16	5-hydroxy-3-(4-methoxyphenyl)-7-[3,4,5-trihydroxy-6-[(3,4,5-trihydroxy-6-methyloxan-2-yl)oxymethyl]oxan-2-yl]oxychromen-4-one	1v2	1v3	1v4	1v5	1v6	1v7	2v4	2v5	2v6	2v7	3v7	4v7	6v7				
17	Apigenin	1v2	1v3	1v4	1v6	2v4	2v5	2v7	3v5	3v7	4v5	4v7	5v6	6v7				
18	Costunolide	1v2	1v3	1v4	1v6	2v3	2v4	2v7	3v6	4v5	4v6	4v7	5v6	6v7				
19	Luteolin 7-rutinoside	1v2	1v6	1v7	2v3	2v4	2v5	2v7	3v6	4v6	4v7	5v6	5v7	6v7				
20	Octinoxate	1v3	1v4	1v5	1v7	2v3	2v4	3v4	3v5	3v6	3v7	4v5	4v6	4v7				

21	1,3-Dicaffeoylquinic acid	1v2	1v3	1v4	1v6	2v3	2v6	3v4	3v5	3v7	4v6	5v6	6v7
22	N1,N5,N10,N14-Tetra-trans-p-coumaroylspermine	1v3	1v6	1v7	2v3	2v6	2v7	3v4	3v5	3v6	3v7	4v6	4v7
23	Acacetin	1v2	1v3	1v4	1v6	2v7	3v5	3v7	4v5	4v7	5v6	6v7	
24	Anisaldehyde	1v2	1v4	1v5	1v6	2v5	3v5	4v5	4v7	5v6	5v7	6v7	
25	Caffeoylquinic acid	1v2	1v3	1v4	1v6	2v3	2v6	3v4	3v5	3v6	3v7	6v7	
26	Hydroquinone	1v2	1v3	1v5	1v6	1v7	2v3	3v4	3v5	3v6	3v7	4v6	
27	Jaceosidin	1v3	1v4	1v7	2v3	2v4	3v5	3v6	4v5	4v6	5v7	6v7	
28	5-O-Caffeoylshikimic acid	1v3	2v3	2v5	2v7	3v4	3v5	3v6	3v7	5v6	6v7		
29	8-{(1S,5R)-4-Oxo-5-[(2Z)-2-penten-1-yl]-2-cyclopenten-1-yl}octanoic acid	1v3	1v6	2v3	3v4	3v5	3v6	3v7	4v5	4v6	4v7		
30	Isochlorogenic acid A	1v3	1v7	2v3	2v7	3v4	3v6	3v7	4v7	5v7	6v7		
31	Linoleoyl ethanolamide	1v3	1v6	2v3	3v4	3v5	3v6	3v7	4v5	4v6	4v7		
32	O-fumarylcarnitine	1v3	1v7	2v3	2v7	3v5	3v6	4v5	4v7	5v7	6v7		
33	Stearidonic acid	1v3	2v3	2v4	3v4	3v5	3v6	3v7	4v5	4v6	4v7		
34	3,4,5-Tricaffeoylquinic acid	1v5	1v7	2v3	2v5	3v6	3v7	4v7	5v6	5v7			
35	HX-3 1	1v2	1v5	2v3	2v6	2v7	3v4	3v5	4v7	5v7			
36	Linarin	1v2	2v4	2v5	2v6	2v7	3v4	3v7	4v7	6v7			
37	Luteolin 7-glucuronide	1v2	1v3	1v5	2v5	3v4	3v5	4v5	5v6	5v7			
38	Tris(2,4-di-tert-butylphenyl) phosphate	1v2	1v3	1v4	1v5	1v6	1v7	2v3	3v4	3v6			
39	2-(hydroxymethyl)-6-[4-[(2S,3S)-3-(hydroxymethyl)-5-[(E)-3-hydroxyprop-1-enyl]-7-methoxy-2,3-dihydro-1-benzofuran-2-yl]-2-methoxyphenoxy]oxane-3,4,5-triol	1v3	1v5	2v3	2v6	3v4	3v6	3v7	5v6				
40	Coumarin	1v6	1v7	2v3	3v4	3v6	3v7	4v6	4v7				

41	2-Naphthaleneacetic acid, decahydro-4-hydroxy-4a-methyl-alpha,8-bis(methylene)-	1v4	1v6	2v4	2v6	4v5	4v7	5v6	6v7
42	3-[(2S,3R,4S,5R,6S)-3,4-dihydroxy-6-methyl-5-[(2S,3R,4S,5S,6R)-3,4,5-trihydroxy-6-(hydroxymethyl)oxan-2-yl]oxyoxan-2-yl]oxy-2-(3,4-dihydroxyphenyl)-5-hydroxy-7-[3,4,5-trihydroxy-6-(hydroxymethyl)oxan-2-yl]oxychromen-4-one	1v7	2v3	2v4	2v7	3v6	4v6	5v7	6v7
43	Luteolin 4'-O-glucoside	1v2	1v6	2v4	2v5	2v6	2v7	3v5	5v6
44	Pheophorbide A	1v3	2v3	2v4	3v4	3v5	3v6	3v7	4v6
45	Quercitrin	1v2	1v6	2v3	2v7	3v4	3v6	4v6	6v7
46	2,5-Furandicarboxylic acid	1v5	2v3	3v4	3v5	3v6	3v7	4v5	
47	Dicoumaroyl Spermidine 1	1v3	1v7	2v3	3v4	3v6	3v7	4v7	
48	Isochlorogenic acid C	1v3	1v5	2v3	3v4	3v6	3v7	5v7	
49	Phthalide	1v3	2v7	3v4	3v5	3v7	4v7	6v7	
50	Sinapaldehyde	1v2	2v4	2v7	4v5	4v6	5v7	6v7	
51	Tributoxyethyl phosphate	1v5	1v6	2v3	3v4	3v5	3v6	3v7	
52	4-Hydroxycoumarin	1v3	2v3	3v4	3v5	3v7	6v7		
53	Chlorogenic acid	1v3	2v3	3v4	3v5	3v6	3v7		
54	Erucamide	1v6	2v3	3v4	3v5	3v6	3v7		
55	Esculamine	1v6	1v7	2v5	4v7	5v6	5v7		
56	Linolenic acid	1v3	2v3	3v4	3v5	3v6	3v7		
57	Monolinolenin (9c,12c,15c)	1v3	2v3	3v4	3v5	3v6	3v7		
58	Rutin	1v6	2v3	2v7	3v6	4v6	6v7		

59	(3R,5S)-4-[(E)-3-(3,4-dihydroxyphenyl)prop-2-enoyl]oxy-1,3,5-trihydroxycyclohexane-1-carboxylic acid	1v3	1v6	2v3	3v4	3v7
60	Acetyltributyl citrate	2v3	3v4	3v5	3v6	3v7
61	Geranitin O-malonyl glucoside	1v5	1v7	4v5	5v6	5v7
62	Hyperin	1v6	2v3	3v6	4v6	6v7
63	Isoquercitrin	1v6	2v3	3v6	4v6	6v7
64	Isorhamnetin 3-robinobioside	1v7	2v7	4v7	5v7	6v7
65	Quercetin-3-O-glucose-6"-acetate	1v2	1v5	1v6	1v7	4v6
66	Quercetin-3-O-rutinosyl-7-O-glucoside	1v6	2v6	3v6	4v6	6v7
67	Syringin	1v3	2v7	3v5	3v7	6v7
68	Adenine	1v7	2v3	3v6	3v7	
69	LPC 18:2	1v4	2v4	4v6	4v7	
70	Bracteatin	2v3	3v6	6v7		
71	Kaempferol-3-O-glucosyl(1-2)rhamnoside	1v5	3v5	4v5		
72	LPC 18:3-1	2v4	4v6	6v7		
73	Luteolin	1v5	4v5	5v7		
74	Reserpine	1v2	2v3	2v6		
75	Sucrose	1v4	2v4	4v6		
76	Cinnamaldehyde	3v7	4v7			
77	L(+)-Arginine	1v3	1v7			
78	LPC 16:0	2v4	4v6			
79	LPC 18:1	1v4	4v7			
80	Luteolin 3',4'-dimethyl ether	1v5	5v7			
81	Methyl 5-oxooctadecanoate	2v3	3v4			

82	Pectolinarigenin	1v4	1v5
83	LPC 18:0	4v7	
84	LPE 18:2	4v7	
85	Quercetin	3v6	

Note: 1v2 means that population 1 compared to population 2, and others the same.
